# The Effectiveness of Sirolimus Treatment in Two Rare Disorders with Nonketotic Hypoinsulinemic Hypoglycemia: The Role of mTOR Pathway

**DOI:** 10.4274/jcrpe.galenos.2020.2019.0084

**Published:** 2020-11-25

**Authors:** Zeynep Şıklar, Tugba Çetin, Nilgün Çakar, Merih Berberoğlu

**Affiliations:** 1 Ankara University Faculty of Medicine, Department of Pediatric Endocrinology, Ankara, Turkey; 2 Ankara University Faculty of Medicine, Department of Pediatric Reumatology, Ankara, Turkey

**Keywords:** AKT2, PTEN, sirolimus, hypoglycemia, treatment

## Abstract

Nonketotic-hypoinsulinemic hypoglycemia (NkHH) is a very rare problem charcterized by increase in glucose consumption without hyperinsulinism. This disorder has mainly been reported in cases with *AKT2* mutation and rarely in cases with *PTEN* mutation. In cases with *PTEN* or *AKT2* mutation, there is no effective therapy other than frequent feeding to counter hypoglycemia. The mammalian target of rapamicin (mTOR) inhibitor, sirolimus, has been used in hyperinsulinemic hypoglycemia that was unresponsive to other medical treatment. In the insulin signaling pathway, both *AKT2* and PTEN function upstream of mTOR. However, the role of Sirolimus on hypoglycemia in *AKT2* and *PTEN* mutations is unknown. Case 1: Six month-old female with *AKT2* mutation [c.49G>A (p.E17K)] and evidence of NkHH. Frequent feeding was unsuccesful in correcting hypoglycemia and her proptosis continued to worsen. Sirolimus treatment was started at three years of age. Subsequently, blood glucose (BG) levels increased to normal levels. Case 2: In a male with PTEN mutation (p.G132V (c.395G>T), persistent NkHH started at 16 years of age (fasting BG: 27 mg/dL, fasting insulin 1.5 mmol/L, while ketone negative). Sirolimus treatment was started and hypoglycemia was succesfully controlled. NkHH is a very rare and serious disorder which is challenging, both for diagnosis and treatment. Additionally, *AKT2* and *PTEN* mutations may result in NkHH. Sirolimus treatment, through mTOR inhibition, appeared to be effectively controlling the peristent hypoglycemia and may be a life-saving therapy in this NkHH due to *AKT2* and *PTEN* mutations.

**What is already known on this topic?**

Nonketotic-hypoinsulinemic hypoglycemia (NkHH) is a very rare problem of glucose consumption increase without hyperinsulinism. In these cases, there is no effective therapy other than frequent feeding to counter hypoglycemia.

**What this study adds?**

Sirolimus treatment may function as a further therapeutic option in NkHH. It could be a lifesaving tool for those kind of disorders as sirolimus appears to control the peristent hypoglycemia effectively in patients with NkHH, by inhibition of mammalian target of rapamicin.

## Introduction

Recurrent/persistent fasting hypoglycema is a life-threatening condition in childhood and is frequently related to either hyperinsulinism or inborn errors of metabolism impairing hepatic glucose production (1). Hyperinsulinism is the most common cause of persistent hypoketotic, hypo-fattyacidaemic, hypoglycaemia in infancy and childhood. In this situation, excessive insulin secretion suppresses the mobilisation of fatty acids from adipose tissue, preventing ketone body synthesis in the liver (2,3). Another well known cause of nonketotic hypoglycemia are fatty acid oxidation defects (4).

Recently, a few cases with unexplained, recurrent and severe fasting hypoglycemia without hyperinsulinism or fatty acid oxidation defects have been reported (1,5,6,7). We prefered to use the term of “nonketotic-hypoinsulinemic hypoglycemia (NkHH)” in these cases.

NkHH is a very rare problem of glucose consumption increase without hyperinsulinism. In 2011, the first case with genetic defects of *AKT2* leading to NkHH was published. *AKT2* is a serine/threonine kinase that plays an important role in insulin signal transduction (7). Normally, when insulin combines with its receptor at target tissue, it requires phosphatidylinositol-3,4,5-trisphosphate (PIP3) to accumulate at the plasma membrane to facilitate insulin transmission within the cell. Gain of function mutation of *AKT2* cause PIP3 accumulation without the need for insulin. The biochemical profile of *AKT2* activating mutation is very similar to hyperinsulinism (7).

Similar to *AKT2* activating mutation, a defect in other molecules that have a role in insulin signalling are expected to be a cause of NkHH. Rarely, NkHH can also develop with mutation of the tumor suppressor gene *PTEN* (8).

Treatment of hypoglycemia in NkHH can be very difficult, because there is no beneficial medical therapy to counteract insulin synthesis or secretion. In these cases, no effective therapy is available other than frequent feeding to prevent hypoglycemia. Thus, there is no available therapy at all in patients who cannot be fed for any reason, including vomiting, gastrointestinal problems, or anorexia.

The mammalian target of rapamycin (mTOR) inhibitor, sirolimus, has been used in hyperinsulinemic hypoglycemia which was unresponsive to other medical treatment (3,9). In the insulin signaling pathway, both *AKT2* and *PTEN* function upstream of mTOR. Thus inhibition of mTOR should counter activating mutations in *AKT2* and *PTEN*. However, the effect of sirolimus in hypoglycemia due to *AKT2* and *PTEN* mutations is unknown.

In this paper, clinical and biochemical characteristics of two rare cases with NkHH are presented. Additionally, the effect of sirolimus on hypoglycemia is reported in these cases.

## Case Reports

### Case 1

A seven month-old female patient was brought to clinic by her family because she had recurrent hypoglycemia for one month. She was born at term with a history of polyhydramnios. On physical examination there was bilateral proptosis, hypertrichosis, hypertelorism, a flat nasal bridge, macroglossia and acanthosis nigricans. At the time of admission her height was 68 cm (50^th^ percentile) with a weight of 7700 g (25-50^th^ percentile) and a head circumference of 44 cm (75^th^ percentile). She had hypoinsulinemia (<0.2 mIU/mL, C-peptide <0.1 ng/mL) and was nonketotic during hypoglycemia when blood glucose (BG) was 27 mg/dL. Other biochemical and hormonal analysis showed normal results. Due to hypoglycemia occuring during fasting, frequent feeding and addition of cornstarch to foods was implemented. Whenever severe hypoglycemia occured, intravenous glucose infusion was also given. 

**Figure 1:**
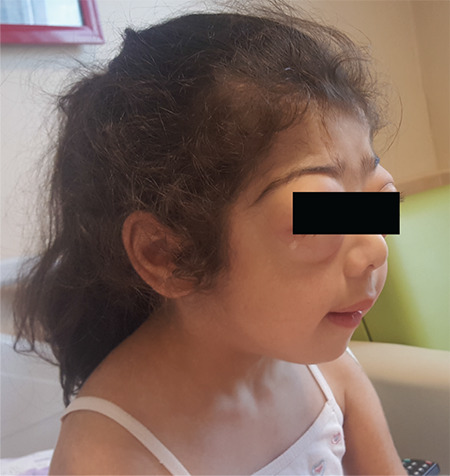
Case 1 with *AKT2* mutation

Genetic analysis revealed a *de novo* *AKT2* mutation [c.49G>A (p.E17K)] in the patient (5). During follow-up, frequent feeding was unsuccesful in treating all the hypoglycemic episodes. Clinically, acanthosis nigricans and proptosis continued to worsen (Figure 1). After informed consent was given by her parents, sirolimus treatment was started at three years of age. On sirolimus treatment BG levels increased to normal levels (mean BG before treatment: 48-52 mg/dL/day, after treatment 77-108 mg/dL/day). Prior to starting sirolimus treatment, it was observed that she could not fast longer than 3 hours although this increased to 4 to 5 hours with the treatment. Neurological evaluation revealed normal language, cognitive, social, and fine motor development with a slight delay in gross motor development.

### Case 2

**Figure 2:**
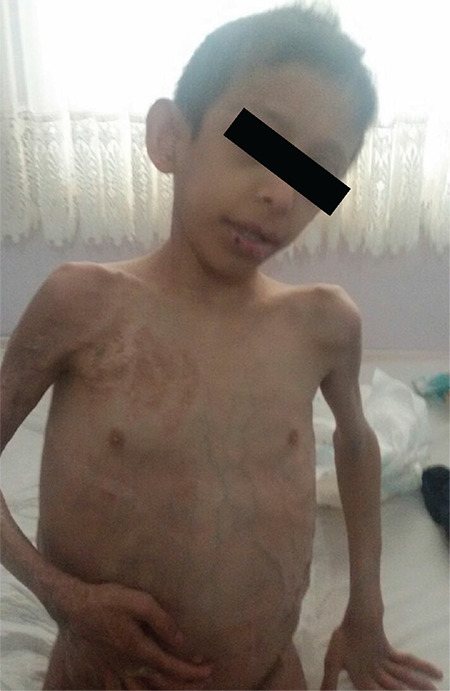
Case 2 with *PTEN* mutation

A male with multiple systemic involvement was diagnosed with hamartoma-tumor syndrome before his admission to the endocrinology department. He had verrucous epidermal nevus and adrenal hemorrhage at birth, and at five month of age renal vein and inferior vena cava thrombosis with hypertension. At 16 months of age, he developed pelvic and retroperitoneal lipomatosis, multiple polyps of the colon and focal segmental glomerulosclerosis. Total colectomy for polyps was caried out due to the recurrent bleeding. He also had macrocephaly, delayed motor mental development and epileptic seizures (Figure 2). These symptoms suggested *PTEN* hamartoma-tumor syndrome (PHTS), so mutation analysis was conducted. Results revealed a *PTEN* mutation [p.G132V (c.395G>T)] (10). Due to the *PTEN* mutation, he carried a high risk of thyroid malignancy. Due to this risk prophylactic thyroidectomy was performed and thyroxine replacement was started.

During his follow-up, at 16 years of age, severe recurrent hypoglycemia (fasting BG: 6 to 27 mg/dL) was noted. Fasting insulin was low (1.5 mmol/L), while ketone was negative. Hypoglycemia was persistent even though there was no known causes of hypoglycemia, with no detection of congenital metabolic disorders.

A tentative diagnosis of NkHH was reached and frequent feeding was offered, but this was ineffective in resolving the hypoglycemic attacks. In addition, feeding was not always possible due to occasional anorectic episodes.

We were aware that *PTEN* may have a role in the insulin signalling pathway and there were reports suggesting that a few patients with *PTEN* mutation also had hypoyglycemic events (7,8).

After informed consent was given, sirolimus treatment was started. Follow-up examinations and evaluations were done at three-monthly intervals. These included complete blood count, serum BUN, creatinine, electrolytes, aminotransferase measurement (aspartate aminotransferase and alanine aminotransferase), lipid profile and hemoglobin A1c. Sirolimus treatment also resulted in an improvement in duration of fasting time in this patient, although the effect was not as marked. Fasting time for case 2 increased to 3 to 4 hours, up from no more than 2 hours, prior to sirolimus. Case 2 required an increased dose of sirolimus during the first month of treatment. With increased dosage, the frequency and severity of hypoglycemia reduced: mean BG before treatment was 46-64 mg/dL/day; after treatment this was 62-92 mg/dL/day. The lowest fasting glucose level of 32 mg/dL was experienced by case 2.

### Sirolimus Dosing

For both patients, initial sirolimus dose was 0.5 mg/m2/day. The dose of sirolimus was then titrated according to the serum level for both patients (between 4 and 12 mg/dL). Final sirolimus dose was 1 mg/m^2^/day in case 1 and 4 mg/m2/day in case 2. Of note, transient leucocytosis was observed in case 1 without any other obvious cause. Duration of sirolimus treatment wase 42 months in case 1 and nine months in case 2.

Informed consent for the publication of these cases was obtained from both sets of parents.

## Discussion

It is accepted that glucose homeostasis is maintained by the action of insulin on muscle, adipose tissue and liver (5). Insulin stimulate energy storage and growth through effects on glucose, lipid and amino acid metabolism. At the cellular level, insulin effects are mediated by a transmembrane tyrosine kinase receptor that phosphorylates insulin receptor substrate (IRS) and other adaptor proteins. Further downstream, insulin signaling leads to activation of AKT serine/threonine kinases (1).

In recent times, NkHH cases due to activation of the insulin signaling pathway have begun to be published. *AKT2* mutation has been shown to be directly causative for this specific condition (1,8,11). *AKT2* is critical for the control of glucose and lipid metabolism. It is recruited to the cell surface by phosphoinositide 3-kinase (PI3K) and phosphorylated by pyruvate dehydrogenase kinase 1 and mTOR c2 kinases. It has a transducer effect during insulin signaling to GLUT4 (12). The causes of hypoglycemia in *AKT2* mutation are related to the activation of the insulin signalling pathway. The first case with a gain of function of *AKT2* causing hypoinsulinemic hypoglycemia was reported in 2011 (1). Since then, a few cases have been reported, including the two presented herein (5,6,7).

*AKT2* is a signal transducer in both glucose metabolism and lipid homeostasis (12). In our case, extra-ocular adipose tissue expansion leading to proptosis was prominent. Some deposits of adipose tissue may be more responsive to *AKT2* mutation than others. The cause of this difference is unknown although one plausible explanation may be different levels of expression of *AKT2* in metabolic tissues (12).

*PTEN* is one the most important tumour suppressors. Deactivation of *PTEN* causes the activation of mTOR c1, which in turn leads to the augmented translocation of specific mRNAs which is crucial for cell growth and proliferation (13). The *PTEN*–PI3K–AKT–mTOR pathway has a central role in the regulation of glucose metabolism. This pathway has downstream effects on the insulin receptor and IRS adaptor molecules. It is known that the PI3K–AKT pathway enhances insulin-mediated glucose uptake and membrane translocation of the glucose transporter GLUT4, and inhibits gluconeogenesis (13). *PTEN* deficiency results in enhanced activation of the AKT signaling pathway. A mutation of *PTEN* may augment PI3K signaling to AKT, which then affects the mTOR pathway (14).

Usually, hypoglycemia in a patient with *PTEN* gene mutation is not noticed. Schmid et al (8) reported a case with PHTS, which was caused by germ line mutations in the *PTEN* gene. The case was treated with sirolimus for uncontrolled tumor cell proliferation. In this case a fasting glucose level of 1.9 mmol/L (reference: 3.6-5.6 mmol/L; equivalent to 35 mg/dL) was detected at the age of 42 months, although there was no extra information about the course of hypoglycemia. In our PTHS patient (case 2), hypoglycemia was detected at 16 years old. Blood sugar levels were very low at times and frequent feeding was ineffective to resolve hypoglycemia. In particular, the management of hypoglycemia in anorectic periods in this patient were very challenging. This situation prompted the search for alternative or additional treatment modalities.

*PTEN* loss induces adipogenic-like transformation in hepatocytes and transcription of genes involved in lipogenesis and β-oxidation (13). Additionally, mTOR has a central role in the regulation of cell cycle and initiation of transcription by translating the signalling for growth and proliferation. The administration of rapamycin (mTOR inhibitor) in patients with *PTEN* mutation has been found to be effective in reducing hamartomatous masses, lipomatous lesions, and thymus hyperplasia with clinical recovery (8,15,16). Hence, both hypoglycemia and uncontrolled cell proliferation in various tissues may be controlled with mTOR inhibition. With sirolimus treatment, hypoglycemia in case 2 was controlled more succesfully.

A dysregulated *PTEN*–PI3K–AKT–mTOR signaling pathway may result not only in extensive tumor cell proliferation, but also deregulation of glucose metabolism. Increased glucose utilization is consistent with hyperactivation of the PI3K/AKT pathway, being one of the key mediators of increased glucose utilization observed in many cancer cells (17). Kinross et al (18) developed a murine model with Pik3ca H1047R mutation. Pik3ca is the gene encoding p110, a catalytic subunit of PI3K. In this model, a dramatic increase in body weight, which was associated with increased organ size, a reduction in BG levels and undetectable insulin levels, were observed. In the human, mosaic activating mutations in PI3K are known to cause segmental overgrowth. In a study which evaluated the metabolic phenotype of 22 patients with mosaic activating mutations affecting PI3K, three patients were found to have early onset, severe, nonketotic hypoglycemia (11).

With all of these findings, it seems probable that dsyregulation of the insulin signalling pathway affecting *AKT2*, *PTEN*, or PI3K could cause NkHH with syndromic features. Unexplained hypoglycemia mimicking that of hyperinsulinism with no detectable insulin should alert the physician to a possible insulin transducing defect. An effective treatment option for hypoglycemia in these patients could be to block insulin signalling. The only known, widely-used agent to impact this pathway is the mTOR inhibitor, sirolimus. Until now, sirolimus has been successfully used for the severe, diffuse form of congenital hyperinsulinism (9). In our cases, we decided to give sirolimus to improve hypoglycemia in a patient with *AKT2* mutation and another patient with PTHS as sirolimus will inhibit mTOR, the next downstream step in *PTEN*/AKT signalling. In both patients hypoglycemia was controlled with sirolimus treatment.

The reported side effects of sirolimus treatment include immunosuppressive effects, oral mucositis, renal dysfunction, pneumonitis, increased serum aminotransferase levels, hepatitis, and dyslipidemia (9,19). Of these, only mild leucocytosis was observed in case 1 without any additional symptoms.

## Conclusion

NkHH is a very rare but significant disorder which presented some challenges in both diagnosis and treatment. Activating mutations in *AKT2* or *PTEN*, upstream from mTOR in the insulin signalling pathway, may lead to NkHH. Sirolimus treatment, resulting in mTOR inhibition, appeared to be effective in controlling the persistent hypoglycemia in two cases. Sirolimus may be a life-saving therapeutic option for some of these rare diseases caused by increased activation of insulin signalling.
